# Neutrophil Extracellular Traps in Dengue Are Mainly Generated NOX-Independently

**DOI:** 10.3389/fimmu.2021.629167

**Published:** 2021-05-26

**Authors:** Fadel Muhammad Garishah, Nils Rother, Silvita Fitri Riswari, Bachti Alisjahbana, Gijs J. Overheul, Ronald P. van Rij, André van der Ven, Johan van der Vlag, Quirijn de Mast

**Affiliations:** ^1^ Department of Internal Medicine and the Radboud Center for Infectious Diseases, Radboud University Medical Center, Nijmegen, Netherlands; ^2^ Center for Tropical and Infectious Diseases (CENTRID), Faculty of Medicine, Diponegoro University, Dr. Kariadi Hospital, Semarang, Indonesia; ^3^ Department of Nephrology, Radboud Institute for Molecular Life Sciences, Radboud University Medical Center, Nijmegen, Netherlands; ^4^ Research Center for Care and Control of Infectious Disease (RC3ID), Universitas Padjadjaran, Bandung, Indonesia; ^5^ Department of Biomedical Sciences, Faculty of Medicine, Universitas Padjadjaran, Bandung, Indonesia; ^6^ Department of Internal Medicine, Hasan Sadikin General Hospital, Faculty of Medicine, Universitas Padjadjaran, Bandung, Indonesia; ^7^ Department of Medical Microbiology, Radboud Institute for Molecular Life Sciences, Radboud University Medical Center, Nijmegen, Netherlands

**Keywords:** neutrophil extracellular traps, NET formation, NADPH-oxidase independent, platelets, dengue, plasma leakage

## Abstract

Neutrophil extracellular traps (NETs) are increasingly recognized to play a role in the pathogenesis of viral infections, including dengue. NETs can be formed NADPH oxidase (NOX)-dependently or NOX-independently. NOX-independent NETs can be induced by activated platelets and are very potent in activating the endothelium. Platelet activation with thrombocytopenia and endothelial dysfunction are prominent features of dengue virus infection. We postulated that dengue infection is associated with NOX-independent NET formation, which is related to platelet activation, endothelial perturbation and increased vascular permeability. Using our specific NET assays, we investigated the time course of NET formation in a cohort of Indonesian dengue patients. We found that plasma levels of NETs were profoundly elevated and that these NETs were predominantly NOX-independent NETs. During early recovery phase (7-13 days from fever onset), total NETs correlated negatively with platelet number and positively with platelet P-selectin expression, the binding of von Willebrand factor to platelets and levels of Syndecan-1. Patients with gall bladder wall thickening, an early marker of plasma leakage, had a higher median level of total NETs. *Ex vivo*, platelets induced NOX-independent NET formation in a dengue virus non-structural protein 1 (NS1)-dependent manner. We conclude that NOX-independent NET formation is enhanced in dengue, which is most likely mediated by NS1 and activated platelets.

## Introduction

Dengue is the most important arboviral infection worldwide, occurring in more than 125 countries (1). Dengue is usually a non-severe illness, but life-threatening complications can occur. The most important complications are a transient vascular permeability syndrome and bleeding (2). The pathogenesis of these complications is still incompletely understood.

Neutrophil extracellular traps (NETs) are web-like chromatin structures released by neutrophils upon exposure to both infectious or non-infectious stimuli. These web-like chromatin structures are decorated with histones and anti-microbial peptides, such as myeloperoxidase (MPO), neutrophil elastase (NE) and cathepsin G (CatG) (3). The primary function of NETs is to capture, immobilize and kill pathogens within the host. However, they can inadvertently result in endothelial and tissue damage, inflammation and increased vascular permeability (4, 5).

NET formation was originally thought to predominantly play a role in the clearance of bacterial infections, but more recent evidence suggest that it is also a feature of some viral infections, including dengue (6–11). However, in dengue virus (DENV) infection, the underlying mechanism of increased NET formation and the association with its complications remain incompletely understood.

NET formation was initially described as a nicotinamide adenine dinucleotide phosphate (NADPH) oxidase (NOX)-dependent pathway (3). More recently, also a NOX-independent pathway was identified (12), in which neutrophils release NETs through blebbing of nuclear membranes rather than undergoing cellular lysis (13, 14). Activated platelets are potent activators of NOX-independent NET formation (15), whereas certain serotypes of bacterial lipopolysaccharide (LPS) and phorbol 12-myristate 13-acetate (PMA) induce NOX-dependent NET formation (3, 16). The coagulation protein von Willebrand factor (VWF) also induces NET formation (17, 18). Notably, platelet activation and increased VWF binding to platelets are features of dengue that contribute to thrombocytopenia (19). We recently developed a serological assay to discriminate NOX-dependent from NOX-independent NET formation and showed previously that in particular NOX-independent NETs are very potent in activating endothelial cells (16).

We postulate that dengue infection is associated with NOX-independent NET formation and that this is associated with platelet activation, endothelial perturbation and vascular permeability. To this end, we investigated the time course of NET formation in both children and adults with dengue infection, determined the contribution of the NOX-independent NET formation and further explored the associations between NET formation with platelet number and activation, binding of VWF to platelets, markers for endothelial perturbation and plasma leakage.

## Materials and Methods

### Patients and Study Design

This study used samples from a prospective observational study performed in Hasan Sadikin General Hospital and Dr. M. Salamun Air Force Hospital, Bandung, West Java, Indonesia. Details of the study have been described elsewhere (19). In summary, a total number of 40 hospitalized acute dengue patients (both children and adults) were enrolled together with 10 healthy controls between January and June 2015. Inclusion criteria were an age of more than one year old, fever (≥ 37.5°C) with clinical symptoms of dengue, thrombocytopenia (platelet count <150 x 10^9^/L), and a positive result of either a dengue NS1 antigen test (PanBio Diagnostics, Windsor, Australia), in-house dengue virus RT-PCR or IgM anti-dengue test (PanBio Diagnostics, Windsor, Australia). Blood samples were drawn during enrolment, hospitalization and approximately two weeks after discharge. Samples were aggregated into groups of days since fever onset, whereby day 1-3 approximated the febrile phase, day 4-6 the critical phase, day 7-13 the early recovery phase and day > 13 the convalescent phase. Plasma leakage was defined by at least an increase in hematocrit of ≥ 20%, or single hematocrit value of >50% for males or >44% for females, or evidence of ascites, pleural effusion and/or gall-bladder wall thickening (GBWT) on ultrasonography.

### Ethics Statement

The study protocol was approved by the Medical Research Ethics Committee, Faculty of Medicine, Universitas Padjadjaran, Hasan Sadikin General Hospital, Bandung, Indonesia (No:04/UN6.C2.1.2/KEPK/PN/2014). Written informed consents were obtained before enrollment of subjects and healthy controls. For children, written informed consents were obtained from parents or legal representatives. All studies were performed in accordance to the Declaration of Helsinki.

### Laboratory Assays Clinical Study

Citrate plasma samples were obtained from 3.2% citrate-anticoagulated blood (BD Vacutainer, Becton Dickinson Biosciences, Franklin Lakes, NJ, USA) centrifuged at 4000 RPM for 15 minutes at room temperature to obtain platelet-poor plasma (PPP). Samples were stored at -80°C for further analysis. Plasma VWF levels were measured by VWF : Ag ELISA using an in-house ELISA, as previously described (19). Syndecan-1 was measured using a human syndecan-1 (CD138) ELISA kit (Abcam, Cambridge, UK). Expression of P-selectin (CD62p) as marker for platelet activation and the binding of VWF to platelets were measured on citrate-anticoagulated whole blood using flow cytometry as previously reported (19). A full blood count was performed using a standard hematology analyzer.

### Isolation of Polymorphonuclear Neutrophils (PMNs)

PMNs were isolated from K2EDTA-anticoagulated whole blood (Becton Dickinson Biosciences, Franklin Lakes, NJ, USA) using Lymphoprep™ density gradient centrifugation (Stemcell Technologies, Vancouver, British Columbia, Canada) as previously described (20). Briefly, whole blood was diluted (2:1 or 1:1) with sterile 1x phosphate-buffered saline (PBS) and centrifuged at 800g for 30 minutes to obtain the PMN fraction. Red blood cells were lysed using sterile distilled-water, and immediately resuspended with 10X PBS. PMNs were resuspended and adjusted to 10^6^ cells/mL with pre-warmed Dubecco’s Modified Eagle Medium/Nutrient Mixture F-12 (DMEM-F12) medium (Thermo Fischer Scientific, Waltham, MA, USA).

### Isolation of Washed Platelets (WPs)

Washed platelets (WPs) were isolated from 3.2% citrate-anticoagulated whole blood (BD Vacutainer, Becton Dickinson Biosciences, Franklin Lakes, NJ, USA) as previously described (21). Briefly, citrate-whole blood centrifuged at 156g without a break for 15 minutes to obtain Platelet-Rich Plasma (PRP). PRP was centrifuged in 330g without a break for 15 minutes with the addition of 1/10 volume acid-citrate-dextrose (ACD) as anticoagulant. Pellets were resuspended in pH 6.5 HEPES Tyrode buffer (HT buffer) and re-centrifuged again in 330g without a break for 15 minutes with the addition of 10ng/mL prostaglandin I_2_ (PGI_2_) (Cayman Chemical, Ann Arbor, MI, USA) to inhibit platelet activation. Pellets were resuspended in pH 7.2 HT buffer. Prior to use, washed platelets were rested for 30 minutes.

### *Ex-vivo* NET Formation Assay

PMNs (1x10^6^ cells/mL) were seeded in flat-bottomed 96-well plates with DMEM/F-12 medium for 1 hour at 37°C until attached. After 1h, cells were washed with pre-warmed PBS and co-cultured with or without washed platelets (ratio 1 PMN *vs*. 50 platelets) in the presence of the following stimuli: 10µg/mL Dengue virus-2 non-structural protein-1 (DENV2 NS1, strain Thailand/16681/84; Native Antigen, Oxford, UK) or 156 µM Thrombin Receptor Activator Peptide 6 (TRAP-6) (Sigma-Aldrich, Zwijndrecht, The Netherlands) for 3 hours at 37°C. For induction of NET formation by patient plasma, 10% plasma was added, together with 20μM D-Phe-Pro-Arg-CMK (PPACK, Santa Cruz Biotechnology, Dallas, TX, USA) to inhibit clot formation. Heat-inactivated DENV2 (0.5x10^7^ median tissue culture infectious dose (TCID)/ml) or mock (medium control) were incubated with PMNs in the presence or absence of autologous washed platelets. Next, adherent NETs were digested with 5U/mL micrococcal nuclease (Worthington Biochemical Corporation, Lakewood, NJ, USA) in fresh DMEM/F-12 medium for 20 minutes at 37°C. NETs in the culture supernatant were measured using the assays as outlined below.

### Quantification of NETs

Quantification of NETs in culture supernatants and patient plasma was performed as previously described (16). In brief, microtiter plates were coated with anti-DNA (DNA-MPO complexes) or anti-histones (H4K8Ac, K12Ac, K16Ac to detect NOX-independent NETs) antibodies, blocked with 2% fish gelatin (Sigma-Aldrich, Schnelldorf, Germany) and incubated with NET-containing samples. Subsequently, anti-MPO antibody (Biolegend, Koblenz, Germany) and horseradish peroxidase (HRP)-conjugated secondary antibody (SoutherBiotech, Birmingham, AL, USA) was added. The absorbance was measured at 450 nm after addition of 3,3’,5,5’-tetramethylbenzidine substrate and sulfuric acid.

### Culture and Preparation of Heat-Inactivated Dengue Virus

DENV2 (strain New Guinea C) was grown on C6/36 cells in Leibovitz’s L-15 medium (Thermo Fisher Scientific, Waltham, MA, USA) supplemented with 10% heat-inactivated FBS (Sigma-Aldrich, Schnelldorf, Germany), 2% tryptose phosphate broth (Sigma-Aldrich, Schnelldorf, Germany), 1× MEM non-essential amino acids (Thermo Fisher Scientific, Waltham, MA, USA) and 50 U/mL penicillin-50 µg/mL streptomycin (Thermo Fisher Scientific, Waltham, MA, USA) at 28°C without CO2. At 6 days post infection the culture supernatant was harvested, centrifuged for 5 min at 1500 g and filtered through an 0.45 μM low protein binding filter (Sigma-Aldrich, Schnelldorf, Germany). Afterwards the medium was transferred over an Amicon Ultra-15 filter with a 100 kDa cutoff (Sigma-Aldrich, Schnelldorf, Germany), which was washed 3 times using Opti-MEM supplemented with GlutaMAX (Thermo Fisher Scientific, Waltham, MA, USA). The concentrated virus on the filter was diluted back to the original volume using Opti-MEM and the purified viral aliquots were stored at -80°C. Viral titers were measured by end-point dilution on BHK-15 cells, using 10 fold dilutions in 96 well plates and scoring for cytopathic effect at 7 days post infection. BHK-15 cells were cultured in DMEM, high glucose (Thermo Fisher Scientific, Waltham, MA, USA) supplemented with 10% heat-inactivated FBS and 50 U/mL penicillin-50 µg/mL streptomycin. Viral stocks were heat inactivated for 30 min at 56°C.

### Statistical Analyses

Data are presented as median value with interquartile range (IQR) unless stated otherwise. Within the dengue patients, differences of parameters in samples from different time points were compared with the Wilcoxon signed rank test. Differences between samples from the dengue patients and the healthy controls were compared with the Mann-Whitney U test. Spearman’s correlation analysis was used for evaluating correlations. For *ex vivo* experiments, we compared differences of NET formation between unstimulated PMNs and stimulated PMNs with different stimuli and in the absence/presence of washed platelets. Differences were analyzed using a one-way ANOVA test with Bonferroni multiple comparisons post-tests. A p-value of <0.05 indicated a significant difference. All analyses were performed using GraphPad Prism 5 (La Jolla, CA, USA) and visualizations were performed using R-Studio version 1.3 for Mac (Boston, MA, USA).

## Results

### Characteristics of Study Participants

A total of 40 hospitalized dengue patients were enrolled together with 10 adult healthy controls. Characteristics of the participants were previously reported (19). In short, the dengue patients had a median (interquartile range; IQR) age of 22 yrs. (17-35 yrs.) and their duration of fever at presentation was 4 days (3-5 days). Three patients were children aged ≤14 years old and twelve patients (30%) were female. Thirty-two (80%) patients were classified as having dengue hemorrhagic fever (DHF) according to the 1997 WHO dengue criteria. Median platelet number at presentation was 62 (34-101) x 10^9^/L and median neutrophil count at presentation was 1.12 (0.64 – 1.81) x 10^9^/L. Samples were grouped according to the sampling day since fever onset; day 1–3 (n = 16), day 4–6 day (n = 33), day 7–13 (n = 21) and day >10 days (n = 16).

### Circulating NETs in Dengue Patients Are Predominantly NOX-Independently-Generated

**Figure 1:**
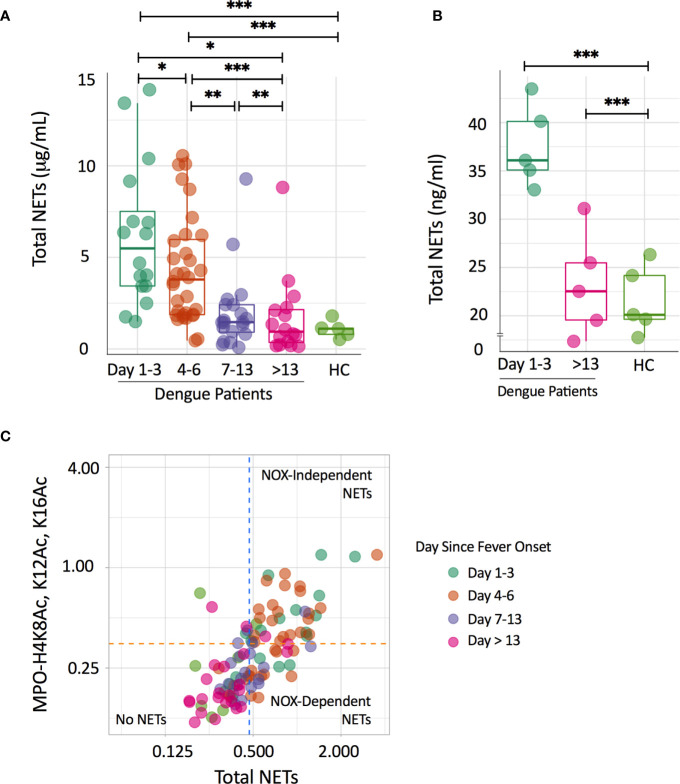
Circulating NETs in dengue patients are mainly NOX-independently generated. **(A)** Time course of total NETs (μg/mL) in plasma of dengue patients according to day since fever onset and in a group of healthy controls (HC). Depicted are individual data together with a box plot showing median with interquartile range. Differences in the dengue patients over time were analyzed using the Wilcoxon signed rank test; differences with the HC group were analyzed using Mann-Whitney U test; *p < 0.05, **p < 0.01, ***p < 0.001. **(B)** NET formation (ng/ml) after incubation of neutrophils with 10% plasma of dengue patients according to day since fever onset and of a group of HC. Depicted are individual data of five plasmas per group tested on 3 neutrophil donors, together with a box plot showing median with interquartile range. Differences with the HC group were analyzed using Mann-Whitney U test;, ***p < 0.001. **(C)** Scatterplot quadrant analysis to differentiate NOX-dependent and NOX-independent NET formation on the basis of results of the total NETs (X-axis) and MPO-H4K8Ac, K12Ac, K16Ac assay (Y-axis). Cut-off points were determined from mean values and standard deviations from HC samples (orange and blue-dotted lines). Samples positive to both assays were classified as NETs derived NOX-independently, while samples only positive for MPO-DNA were classified as NETs derived from NOX-dependently. HC, healthy controls; NOX, NADPH-oxidase.

We found that levels of NETs were higher in plasma samples of dengue patients in the early phases of infection compared to samples from the later phases and samples of healthy controls (**Figure 1A**). Median concentrations of total NETs were 5.5 μg/ml (3.4 - 8.6 μg/ml) at day 1-3, 3.9 μg/ml (1.9 - 6.1 μg/ml) at day 4-6, 1.5 μg/ml (0.8 - 2.4 μg/ml) at day 7-13 and 0.9 μg/ml (0.2 - 2.2 μg/ml) at day > 13. In adult healthy controls, NETs were only detected in 5/10 subjects with a median value of 1.1 μg/ml (0.6 - 1.5 μg/ml). There were no significant differences in concentrations of total NETs between males and females and patients below and above 18 years old (data not shown). Concentrations of total NETs correlated positively with neutrophil number in the early (day 1-3) phase of dengue (*r_s_*=0.56, *p=*0.027). Additionally, plasma of dengue patients at day 1-3 was able to induce NET formation *in vitro* in isolated neutrophils, whereas plasma of patients at day>13 and of healthy controls did not induce NET formation (**Figure 1B**).

Next, we evaluated whether the increased NETs were NOX-dependently or NOX-independently generated. Using a scatterplot quadrant analysis (**Figure 1C**), samples from the early phases of dengue (day 1-3 and day 4-6) were generally positive in both the total NETs and MPO-H4K8Ac, K12Ac, K16Ac ELISA (NOX-independent NETs), suggesting that NETs were predominantly NOX-independently generated (16). Notably, NETs in samples from the later phases of dengue (day 7-13; day >13) and from healthy controls largely showed no NETs or fell in the quadrants fitting a NOX-dependent NETs. Taken together, we show that NETs in dengue patients are predominantly NOX-independently generated.

### Role of Platelets in NOX-Independent NET Formation

**Figure 2:**
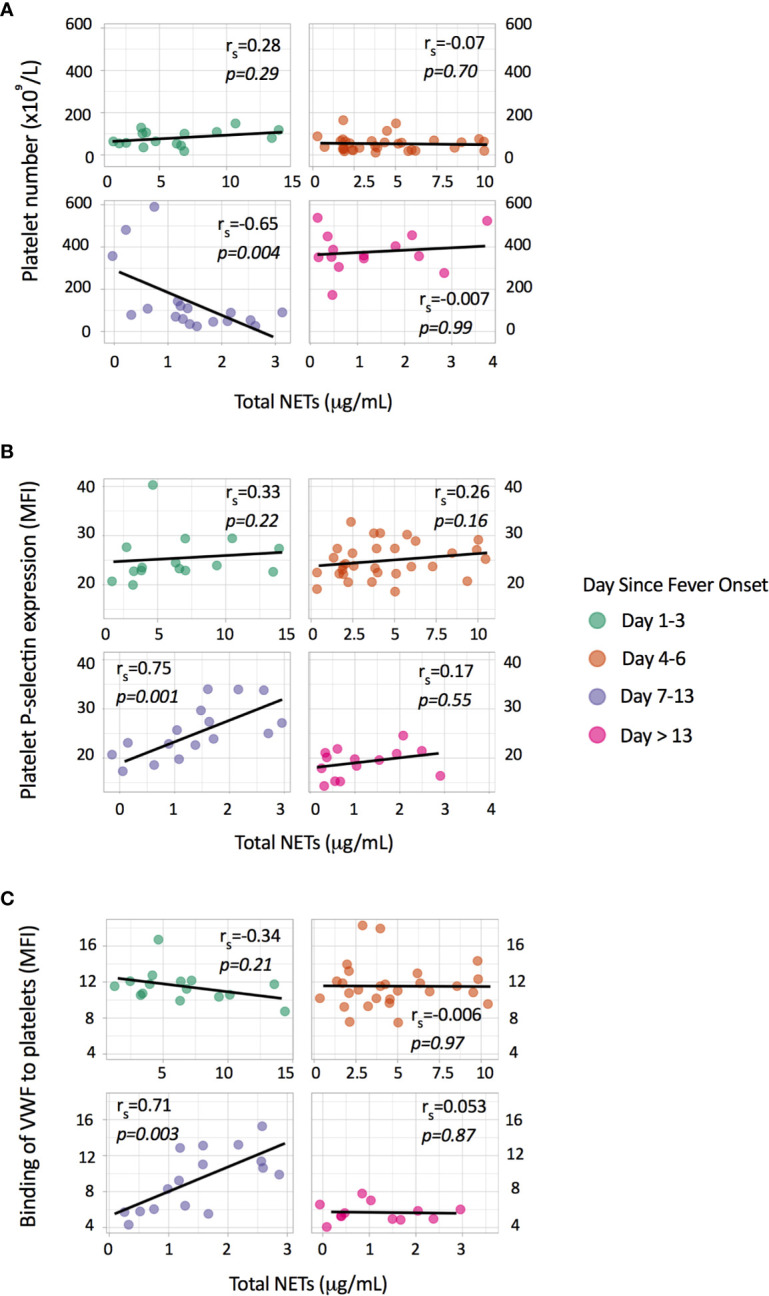
Associations of total NETs with platelet parameters. Depicted are individual data points with the Spearman correlation between total NETs (μg/mL) and **(A)** platelet number (x10^9^/L), **(B)** platelet P-selectin expression (MFI) and **(C)** binding of von Willebrand factor (VWF) to platelets (MFI). Platelet P-selectin expression and binding of VWF to platelets were measured by flow cytometry and are expressed as mean fluorescence intensity (MFI).

Activated platelets stimulate NOX-independent NET formation (3, 13). Soluble P-selectin and platelet-derived microparticles that are generated during platelet activation also induce NOX-independent NET formation (22, 23). Thrombocytopenia and platelet activation are features of symptomatic dengue infections and we postulated that platelets play a substantial role in NOX-independent NET formation in dengue. We first determined associations between platelet parameters and total NETs in our cohort. We observed a significant negative correlation between platelet number and total NETs on day 7-13 (*r_s_*=-0.65, *p*=0.004), but not in the earlier phases of dengue (**Figure 2A**). Platelet P-selectin expression on the platelet membrane, which is a specific marker for platelet activation, and the binding of VWF to platelets showed a strong positive association at day 7-13 (*r_s_=*0.75, *p=*0.001 for P-selectin and *r_s_=*0.71, *p=*0.003 for VWF), but not at the earlier phases (**Figures 2B, C**).

**Figure 3:**
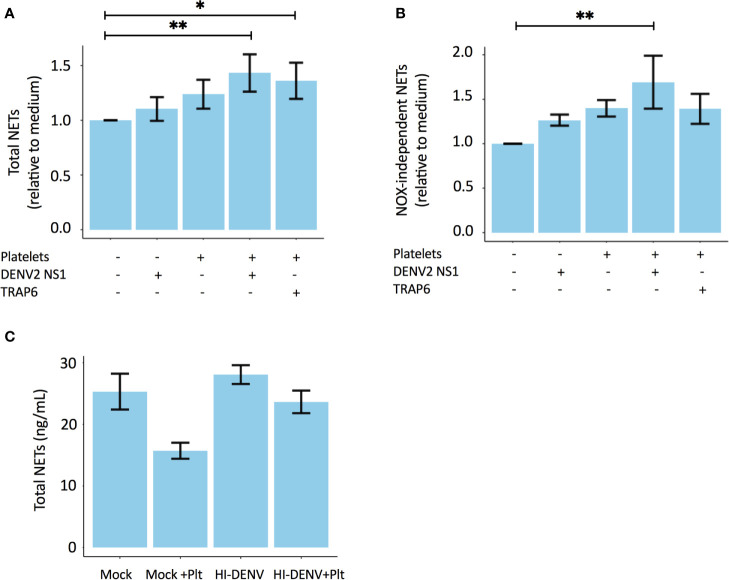
Platelets stimulate NOX-independent NET formation in a DENV NS1-dependent manner. Polymorphonuclear neutrophils (PMNs) of healthy volunteers were co-incubated with autologous platelets in the presence or absence of 10 µg/mL DENV2 NS1 or 156μM platelet agonist TRAP-6. Experiments were performed in triplicate with 2 donors for each condition. Depicted are the relative values to medium (unstimulated samples) for **(A)** Total NETs and **(B)** NOX-independent NETs. **(C)** Heat-inactivated DENV2 does not induce NET formation. PMNs isolated from three healthy donors were incubated with 0.5x10^7^ TCID/mL heat-inactivated DENV2 or medium control (mock) in the presence or absence of autologus washed platelets (Plt). Depicted is the total NET formation (ng/mL). Data are presented as mean with standard error mean (SEM) and analyzed using repeated measure a one-way ANOVA and Bonferroni’s multiple comparisons test; *p < 0.05, **p < 0.01. TCID, Tissue culture infectious dose.

Next, to further validate the involvement of platelets in NOX-independent NET formation, we stimulated primary human polymorphonuclear neutrophils (PMNs) from healthy volunteers with dengue virus 2 non-structural protein 1 (DENV2 NS1) in the presence or absence of autologous washed platelets (WPs). Notably, DENV2 NS1 is known to activate platelets (24). Our data reveal that the combination of both DENV2 NS1 and activated platelets are the most potent in inducing NET formation (**Figure 3A**), in particular NOX-independent NETs (**Figure 3B**). Stimulation of PMNs using heat-killed DENV2 in the presence or absence of autologous platelets did not induce NET formation (**Figure 3C**). This could be due to the heat inactivation, which might jeopardize dengue virus induced activation of neutrophils.

### NETs and Vascular Permeability

**Figure 4:**
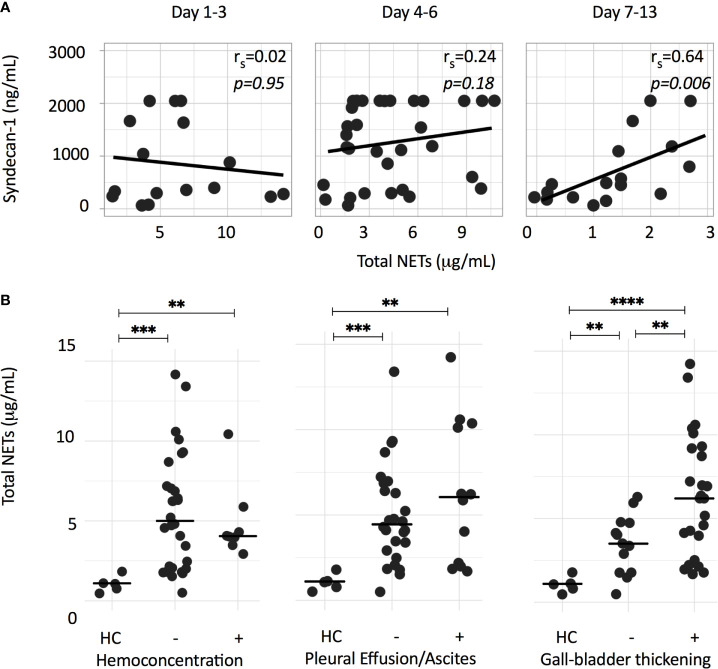
NET formation is associated with syndecan-1, as a surrogate marker for glycocalyx damage, and markers of plasma leakage. **(A)** Depicted is the Spearman correlation between total NETs (μg/mL) and plasma syndecan-1 concentrations (ng/mL) at different time points. **(B)** Total NETs (μg/mL) of dengue patients from either acute or critical phase were grouped according to the presence of hemoconcentration, pleural effusion/ascites or a thickened (>3 mm) gall bladder wall. Hemoconcentration was defined as an increase in hematocrit of ≥ 20%, or single hematocrit value of >50% for male or >44% for female patients. Differences were assessed using Mann-Whitney U test. **p < 0.01, ***p < 0.001, ****p < 0.0001. HC, healthy controls.

We previously showed that NOX-independent NETs have strong immunostimulatory effects on the endothelium, leading to vascular leakage in an *in vitro* assay (16). Notably, a transient endothelial dysfunction with disturbances of the endothelial glycocalyx layer and plasma leakage are features of severe dengue (25, 26) and we postulate that NETs contribute to this transient vascular permeability syndrome. We therefore explored associations of plasma NET concentrations with plasma concentrations of the endothelial activation marker VWF and the glycocalyx marker syndecan-1. The glycocalyx is a layer of membrane-attached macromolecules that covers the luminal surface of endothelial cells and plays a key role in the endothelial barrier function. Syndecan-1 is a component of the glycocalyx and shedding of syndecan-1 is used as a surrogate marker for glycocalyx damage. Plasma syndecan-1 were positively associated with total NETs on day 7-13 (*r_s_=*0.64, *p<*0.01) (**Figure 4A**), but not with plasma VWF (data not shown).

Next, we assessed the associations of total NETs measured in either acute or critical phase samples with parameters of plasma leakage. With the exception of a significantly higher concentration of total NETs in patients with a thickened gall bladder wall, no significant differences were observed for patients with and without hemoconcentration, pleural effusion or ascites (**Figure 4B**).

## Discussion

The role of NET formation in dengue infection is increasingly recognized. Here, we provide novel insights in the NET formation in dengue patients. NETs can be derived NOX-dependently or NOX-independently. NOX-dependent NETs are characterized by cleavage of the histone N-terminal tails, whereas in NOX-independent NETs, histone N-terminal tails are preserved (27). Using a NET assay that detect unmodified N-terminal histone tails on NETs (16), we show that NET formation in dengue is predominantly NOX-independent.

To the best of our knowledge, only one previous study reported on the presence of circulating NETs in patients with dengue (10). Opasawatchai et al. showed that patients with dengue hemorrhagic fever had elevated levels of NETs. In addition, these authors showed that NETs decreased DENV infectivity in an *in vitro* assay. Their findings that NET formation in dengue was independent of peptidylarginine deiminase type 4 (PAD4)-mediated histone H3 hyper-citrullination also supports our present finding that NET formation in dengue predominantly occurs NOX-independently, as citrullination is a feature of NOX-dependent NETs.

NOX-independent NET formation is a rapid process in which neutrophils retain their capacity to phagocytose and degranulate (13). Activation of the NOX-independent pathway requires co-stimulatory signals from both an antigen and a secondary signal, such as activated platelets (13, 15). There is consensus that activated platelets mediate NOX-independent NET formation (3, 15, 23, 28). Thrombocytopenia and platelet activation are prominent features of dengue (24), and we confirmed in our present study that platelets have the capacity to promote NOX-independent NET formation in a DENV2 NS1-dependent manner. DENV2 NS1 has strong immune properties, including the activation of platelets (24). DENV2 NS1 stimulation in the absence of platelets did not result in NET formation. Interestingly, Sung et al. recently identified the C-type Lectin receptor-2 (CLEC2) platelet receptor as an important factor of lethal dengue virus infection (11). Activation of CLEC2 by DENV led to the formation of platelets-derived microparticles, which promoted the formation of NETs *via* crosstalk with C-type Lectin Domain 5A (CLEC5A) and Toll-like receptor 2 (TLR2) on neutrophils. Other mechanisms have also been implicated in the activation of platelets in dengue, including cell-free histones (29), thrombin and mast cell-derived serotonin and tryptase (30). In addition, platelet activating factor (PAF), which is known to be increased in dengue (31) and lipopolysaccharide (LPS), which has been reported to be increased as a result of loss of intestinal integrity (32), were both implicated as triggers of NET formation and may, therefore, also play a role in the pathogenesis of dengue (20, 33). Finally, because NET formation was already observed early during dengue in the febrile phase, we cannot exclude that products, like histones, released during NET formation may contribute to plasma leakage.

We assessed correlations of total NETs with platelet and endothelial parameters, but only found significant correlations in samples taken at day 7-13 of illness, when the patients were generally in the convalescent phase of the infection. In our opinion, this does not preclude a role for platelets in NET formation on the one hand, and a role for NETs in vascular leakage on the other hand. Possible explanations for this seeming discrepancy include the following. First, the acute phases of dengue are characterized by major distortions in the intravascular blood compartment, which may mask possible correlations at this time point. Second, in hospitalized patients with dengue, platelets are uniformly affected in the febrile/critical phases with marked thrombocytopenia and platelet activation. The etiology of thrombocytopenia is multifactorial and not restricted to platelet activation. In addition, P-selectin expression on the platelet membrane may not fully capture the activation status of platelets at this time point. This is also suggested by the fact that P-selectin expression at day 7-13 was similar as at day 1-3 and day 4-6. Third, the platelet effects may be caused by platelet-derived microparticles or platelet-derived proteins, such as platelet factor-4, that were not measured in this study (34, 35).

Another important question is whether NET formation during dengue is beneficial or detrimental for the host (36). On the one hand, NETs are capable of containing pathogens, thereby preventing further dissemination within the host. Previously, NETs were shown to function as viral-traps that actively capture human immunodeficiency virus viral-like particles (HIV-VLP) (9). In addition, the myeloperoxidase (MPO) and α-defensin components of NETs mediate antiviral activity and NETs were shown to reduce DENV infectivity *in vitro* (9, 10). NETs may also have detrimental effects on the endothelium and play a role in intravascular coagulation and microvascular perfusion defects (16, 37). Endothelial dysfunction with plasma leakage, coagulopathy and platelet dysfunction are a hallmark of severe dengue infections. Recent data on the role of NETs in the pathogenesis of pulmonary edema in influenza and Covid-19 highlight the possible detrimental effects of NETs on the endothelium and coagulation (4, 6, 38). We found a strong correlation between NET formation and plasma syndecan-1 at day 7-13 of illness, which further suggests endothelial glycocalyx perturbation. NETs constituents such as neutrophil elastase, MPO and histones are cytotoxic towards endothelial cells (5, 39, 40) and have been linked to kidney injury in hantavirus infection (8). MPO was also demonstrated to induce syndecan-1 shedding through cationic charge changes (41), whereas whole NETs might jeopardize endothelial cell tight junctions and induce reorganization of endothelial cytoskeleton (39, 42). In our present study, we observed significantly higher levels of NETs in patients with thickening of the gall bladder wall, but no differences in NETs between those with and without hemoconcentration or ascites/pleural effusion. This may seem at odds with an important role for NOX-independent NETs in severe plasma, despite the fact that we have previously shown *in vitro* that NOX-independent NETs are potent in activating endothelial cells (16). There is a wide spectrum of manifestations of vascular pathology in dengue and subclinical plasma leakage is common in patients with even mild disease. In addition, the pathogenesis of severe plasma leakage that results in hemoconcentration or ascites/pleural fluid is a complex multifactorial process, involving an interplay of different host immune, endothelial and viral factors. In our opinion, this may explain why severe plasma leakage was not seen in some patients with higher NETs concentrations.

Nevertheless, we acknowledge that our study does not allow to draw firm conclusions on the causal role of NETs in the complications of dengue, and the possible role of platelets in NET formation. The absence of a correlations between platelet parameters and NETs in the early phases of dengue may even argue against such a causative role, as outlined above. In addition, platelets can attach to different leukocytes in the circulation, including neutrophils. These complexes can be assessed using flow cytometry. Previous studies in dengue patients have reported both increased (43) as well as decreased (44) platelet-neutrophil complex formation. Unfortunately, in our present study, platelet-neutrophil complexes were not assessed.

In conclusion our results show that NETs in dengue patients are predominantly NOX-independently generated, in which both DENV2 NS1 and activated platelets may play an important role.

## Data Availability Statement

The raw data supporting the conclusions of this article will be made available by the authors, without undue reservation.

## Ethics Statement

The studies involving human participants were reviewed and approved by Medical Research Ethics Committee, Faculty of Medicine, Universitas Padjadjaran, Hasan Sadikin General Hospital, Bandung, Indonesia. Written informed consent to participate in this study was provided by the participants’ legal guardian/next of kin.

## Author Contributions

FG, NR, JV, and QM designed the experiments, analyzed the data and wrote the manuscript. FG and NR performed the experiments. SF, BA, and QM provided the patient samples, clinical data and performed the platelet activation measurements and the ultrasonography examinations. GO and RR provided DENV2 and revised the manuscript. QM, JV, and AV supervised the study. All authors contributed to the article and approved the submitted version.

## Funding

FG is financially supported by the Indonesian Endowment Fund for Education (LPDP) Scholarship from the Ministry of Finance Republic of Indonesia. NR was supported by the Radboud Institute of Molecular Life Sciences (RIMLS) PhD-program.

## Conflict of Interest

The authors declare that the research was conducted in the absence of any commercial or financial relationships that could be construed as a potential conflict of interest.
